# Interleukin-6 in blood and bronchoalveolar lavage fluid of hospitalized children with community-acquired pneumonia

**DOI:** 10.3389/fped.2022.922143

**Published:** 2022-09-08

**Authors:** Yun Zhang, Wenyu Zheng, Haonan Ning, Jing Liu, Fuhai Li, Xiuli Ju

**Affiliations:** ^1^Department of Pediatrics, Qilu Hospital of Shandong University, Jinan, China; ^2^Department of Biostatistics, School of Public Health, Cheeloo College of Medicine, Shandong University, Jinan, China

**Keywords:** interleukin-6, bronchoalveolar lavage fluid, C-reactive protein, erythrocyte sedimentation rate, community-acquired pneumonia, children, disease severity, pathogen detected

## Abstract

**Background:**

Host biomarkers and cytokines help in the prediction of disease severity in adults with community-acquired pneumonia (CAP). Accurate assessment of pathogens and disease severity is essential to clinical decision-making. There are few validated prognostic tools in blood and bronchoalveolar lavage for children with CAP to assist with proper decision and management.

**Methods:**

We performed a retrospective study of 118 children under 18 years of age, hospitalized for CAP with bronchoalveolar lavage management within the first 2 days. The primary outcome was disease severity: mild (with no complications), moderate (with mild to moderate complications), and severe (with severe complications). Comparison and performance analysis of biomarkers and cytokines in the blood or bronchoalveolar lavage fluid (BALF) across different severity categories/different pathogens were performed.

**Results:**

Analysis of 118 CAP cases revealed significant differences in the BALF levels of IL-6 (*p* = 0.000), CRP (*p* = 0.001), and ESR (*p* = 0.004) across different severity categories, while BALF IL-6 level was indicated as the best indicator to discriminate mild from moderate-to-severe cases with highest AUC (0.847, 95% CI: 0.748–0.946), fair sensitivity (0.839), and specificity (0.450), and severe from non-severe cases with highest AUC (0.847), sensitivity (0.917), and specificity (0.725). ALL biomarkers and cytokines exhibited no significant differences across different pathogen categories (*p* > 0.05), while BALF IL-6 (*p* = 0.000), blood ANC (*p* = 0.028), and ESR (*p* = 0.024) levels were obviously different in comparison to single Mycoplasma pneumoniae (MP)-, bacteria-, or virus-positive group vs. non-group. Blood CRP (*r* = 0.683, *p* = 0.000) and ESR (*r* = 0.512, *p* = 0.000) levels revealed significant correlation with the hospitalization course (HC). Among all the BALF cytokines, only BALF IL-6 showed a significant difference (*p* = 0.004, *p* < 0.01) across different severity categories, with good performance for predicting CAP severity in hospitalized children (AUC = 0.875, *P* = 0.004). Blood IL-6 and BALF IL-6 levels showed no significant correlation; in addition, BALF IL6 was better at predicting CAP severity in hospitalized children (AUC = 0.851, *p* = 0.011, *p* < 0.05) compared to blood IL-6.

**Conclusion:**

BALF IL-6 and blood CRP levels, and ESR may have the ability for discriminating disease severity in hospitalized children with CAP, whereas WBC count and ANC have limited ability. No biomarkers or cytokines seemed to have the ability to predict the pathogen category, while BALF IL-6, blood ANC, and ESR may assist in the diagnosis of single MP, bacteria, and virus infections, respectively.

## Introduction

Community-acquired pneumonia (CAP) is the leading cause of death in children < 5 years of age worldwide. It is also one of the most frequent infectious diseases in children, leading to large antibiotic use and hospitalization even in industrialized countries (1). Biomarkers and cytokines reflecting the host’s response to an infection offer an objective measure of disease severity that may improve prognostication of children with CAP. Accurate assessment of pathogens and disease severity is essential to clinical decision-making. There are a few validated prognostic tools in blood, not to mention in bronchoalveolar lavage, in children with CAP to assist with proper decision and management.

The white blood cell (WBC) count and absolute neutrophil count (ANC) are often elevated in children with CAP; however, WBC count and ANC were not associated with disease severity in one study on children with CAP (2). C-reactive protein (CRP) is an acute-phase reactant produced in response to interleukin-6 (IL-6), which is an inflammatory cytokine. Erythrocyte sedimentation rate (ESR) involves the measurement of the rate at which the erythrocytes sediment inside a test tube, which reflects the dynamics of blood condition with ions, cytokines, hemoglobin, and other compounds. Both CRP and ESR have been studied as tools for differentiating viral from bacterial etiology; however, less work has been done to understand their prognostic abilities.

In 1973, researchers at Osaka University led by Tadamitsu Kishimoto first reported that a soluble factor secreted by T cells was important for antibody production by B cells; subsequently, this soluble factor was cloned as IL-6, which turned out to have various roles in several autoimmune diseases (3, 4). IL-6 in blood and bronchoalveolar lavage fluid (BALF) has been verified to predict bad outcomes in hematological malignancy pathogen prognosis ability (5). Studies in adults with CAP have revealed that these markers are associated with disease severity (6–8), but data in children remain limited.

Sometimes, severe CAP cases showed no significantly abnormal biomarker levels, ending up with clinicians’ ignoring the underlying poor outcomes. Hence, validated good predictors for the prognosis of sensitive severity factors in the early phase of CAP were of precious value.

Our study aims to investigate the biomarkers and cytokines of blood and BALF in hospitalized children with CAP, and analyze the association between these indicators and CAP severity, pathogen detected, or hospitalization course (HC) in a retrospective cohort.

## Materials and methods

This study was a retrospective study of children aged under 18 years who were hospitalized for CAP and had bronchoscopy lavage management within the first 2 days after hospitalization. Each case was diagnosed based on the clinical manifestations and chest radiograph findings. The study was approved by the Qilu Hospital of Shandong University Institutional Review Board.

### Inclusion/exclusion criteria

For this study, we enrolled 118 children aged under 18 years with manifestations and signs of CAP from August 2018 to December 2021, which had focal findings on a CXR or CT scan diagnosed as CAP (defined as CXRs or CT scans with typical pneumonia radiography signs documented by the radiologist during hospitalization) and had bronchoscopy lavage management within the first 2 days of HC. CAP was diagnosed based on the presence of more than one of the following symptoms: new or different cough or sputum production, chest pain, dyspnea, tachypnea, or abnormal auscultatory findings (9). All children were eligible for the indications of Guideline of Pediatric Flexible Bronchoscopy in China (2018 version): (1) Laryngeal stridor; (2) recurrent or persistent wheeze; (3) local stridor; (4) chronic cough of unknown cause; (5) recurrent respiratory tract infection; (6) suspicious foreign body aspiration; (7) hemoptysis; (8) difficulty in weaning mechanical ventilation; and (9) abnormal imaging results in lungs, such as (1) dysplasia or malformation in trachea or bronchi; (2) atelectasis; (3) emphysema; (4) mass lesions of lungs; (5) diffuse lesions of lungs; (6) mediastinal emphysema; (7) space occupying focus of mediastinum or airway; (8) dysplasia of blood vessels, lymphangion, or esophagus; (9) differentiating diagnosis of lesions in pleural cavity; (10) pathogenic diagnosis and treatment of infections in lungs; (11) thoracic trauma with suspicious airway rupture; (12) interventional therapy with bronchoalveolar lavage; (13) assessment and management of airways in perioperative period; (14) assistance of endotracheal intubation and gastric intubation; and (15) other conditions for differentiating diagnosis. In addition, all cases underwent bronchoalveolar lavage until being excluded by contraindications (such as severe cardiopulmonary hypofunction, severe arrhythmia, high fever, severe hemoptysis, and severe malnutrition). In every child, bronchoscopy is performed under a reasonable and proper type of sedation or anesthesia to ensure safety. We excluded children hospitalized within less than 2 weeks before this time of hospitalization, those with a history of aspiration or aspiration pneumonia, those predisposed to glucocorticoids, and/or those with immunocompromised or chronic medical conditions that predisposed to severe or recurrent pneumonia (e.g., immunodeficiency, chronic corticosteroid use, chronic lung disease, malignancy, sickle cell disease, congenital heart disease, patients dependent on tracheostomy, and neuromuscular disorders impacting respiration). Children enrolled within 30 days before the hospitalization were excluded to ensure a distinct infection episode.

### Information collection

Demographic, historical information, clinical signs and data, and severity discriminating points were obtained from the medical record. All cases had blood, urine, and/or nasopharyngeal swabs collected at the time of hospitalization.

### Outcome measurements

Mild cases were defined as typical CAP manifestations without complications or poor outcomes. The moderate cases were classified as hospitalization with at least one of the following symptoms: (1) Respiratory rate higher than WHO classification for age; (2) apnea; (3) increased work of breathing (e.g., retractions, dyspnea, nasal flaring, and grunting); (4) PaO2/FiO2 ratio > 250; (5) multilobar infiltrates; (6) PEWS score; (7) altered mental status; (8) hypotension; (9) presence of effusion; (10) comorbid conditions (e.g., Hemoglobin SS disease, immunosuppression, and immunodeficiency); and (11) unexplained metabolic acidosis. Severe cases required the presence of at least one of the following symptoms: (1) invasive mechanical ventilation; (2) fluid refractory shock; (3) acute need for non-invasive positive pressure ventilation; (4) hypoxemia requiring FiO2 greater than inspired concentration or flow feasible in general care area; and (5) death (10).

### Biomarker measurements

The WBC count, ANC, and ESR assays were performed on the CELL-DYN Sapphire (Abbott Diagnostics, Lake Forest, IL). CRP assays were performed on the Dimension Vista 1500 (Siemens Medical Solutions United States, Inc., Malvern, PA) with a functional sensitivity of 0.29 mg/dL. The concentration of cytokines in the blood and BALF was measured at the Center for Clinical Research of Qilu Hospital of Shandong University. Cytokines were tested by multiplex microsphere flow immunofluorescence assay. All the reagents are naturally brought to room temperature before use. (1) Preparation of the microsphere: Before the microsphere preparation experiment, vortex the microspheres for 30 s, blow gently with a pipette about 30 times, and add the sample immediately. (2) Preparation of washing buffer: Return the 10X washing buffer to room temperature. After all salts are dissolved, dilute to 1X with deionized water for use. The steps are as follows: (1) add 25μL of assay buffer to the sample tube; (2) add 25μL of sample to the sample tube: (3) add 25μL of detection antibody to the sample tube; (4) add 25μL of capture microsphere antibody to the sample tube; (5) incubate for 2 h at room temperature (25 ± 1°C) in the dark with shaking (400–500 r/min); (6) add 25μL of SA-PE to the sample tube; (7) incubate for 0.5 h at room temperature (25 ± 1°C) in the dark with shaking (400–500 r/min); (8) add 1,000μL of 1 × washing buffer to each tube, vortex for a few seconds, centrifuge at 300–500 g for 5 min, slowly pour out the liquid, and place it upside down on an absorbent paper; (9) add 150–300μL of wash buffer to each tube according to the flow cytometer loading requirements; and (10) vortex for 10 s to resuspend the microspheres and test immediately. Biomarker and cytokine measurements less than the limit of detection were replaced with estimates equal to the limit of detection divided by the square root of 2.

### Statistical analysis

Statistical analysis was performed using SPSS, version 25 (SPSS Inc., Chicago, IL, United States). The median biomarker or cytokine concentrations with 25th and 75th percentiles were reported. Differences across every two groups were tested by using the Mann–Whitney *U*-test. Differences across severity groups and pathogen categories were tested by using a Kruskal–Wallis test. The correlation of biomarkers or cytokines with the HC was tested by Spearman correlation analysis. For continuous data, including biomarkers and cytokine levels, receiver operating characteristic (ROC) curves analyses were performed, and area under the curve (AUC) values are presented including 95% confidence intervals (95% CIs) (*p*-values were not corrected for multiple comparisons). Optimal cut-offs for cytokines discriminating in severe vs. non-severe patients or mild vs. moderate–severe patients were calculated using Youden’s index. A two-sided *p*-value < 0.05 was taken as the cut-off for statistical significance.

## Results

A total of 118 children were eligible to be enrolled in our study, and all had blood WBC, ANC, CRP, ESR, and BALF interleukin 6 (BALF IL-6) tested. Among these children, 28 children had been tested for all the cytokines in BALF, such as IL-1β, IL-2, IL4, IL-6, IL-10, IL-12p70, IL-17, TNF-α, and IFN-γ; among the remaining 90 cases, 25 had both blood and BALF IL-6 levels tested.

### Characteristics of all the 118 cases

Of the entire enrolled 118 children, the mean age was 7.2 years, the mean weight was 25.0 kg, and 60.0% of children were boys. Of these 118 cases, 81 (68.6%) had mild CAP, 24 (20.3%) had moderate CAP, and 13 (11.1%) had severe CAP, and there were no significant differences across the three severity categories in age (*p* = 0.340), sex (*p* = 0.492), and weight (*p* = 0.209). Among these cases, we had 17 (14.4%) virus-positive reports, 34 (28.8%) Mycoplasma (MP)-positive reports, 22 (18.6%) bacteria-positive reports, and 56 (47.5%) cases had no positive pathogen reported. Thirteen (11.0%) cases had only virus detected, 24 (20.3%) had only MP detected, 13 (11.0%) had only bacteria detected, 2 (1.7%) had both virus and MP detected, 1 (0.8%) had both virus and bacteria detected, 7 (5.9%) had both MP and bacteria detected, and 1 (0.8%) had the virus, MP, and bacteria all detected (Table 1).

**TABLE 1:** Characteristics of entire cohort of 118 cases.

	*N* = 118
**Age, y, mean (SD)**	7.2 (9.8)
**Weight, kg, mean (SD)**	25.0 (12.4)
**Males, *n* (%)**	60 (49.8)
**Fever, *n* (%)**	89 (75.4)
**Cough, *n* (%)**	102 (86.4)
**Complication, *n* (%)**	
Atelectasis	15 (12.7)
Hydrothorax	22 (18.6)
Pneumothorax	1 (0.8)
Sepsis, *n* (%)	13 (11.0)
**Pathogen detected, *n* (%)**	**62 (52.5)**
Virus*, *n* (%)	**17 (14.4)**
Influenza	10 (8.5)
Coronavirus	5 (4.2)
Human metapneumovirus	5 (4.2)
Rhinovirus	4 (3.4)
Epstein-Barr virus	1 (0.8)
**Mycoplasma pneumoniae, *n* (%)**	**34 (28.8)**
Typical bacteria**, *n* (%)	**22 (18.6)**
Streptococcus pneumoniae	12 (10.2)
Haemophilus influenzae	10 (8.5)
Staphylococcus aureus	3 (2.5)
**Disease severity, *n* (%)**	
Mild	81 (68.6)
Moderate	24 (20.3)
Severe	13 (11.1)

*Only common viruses are listed in this table. **Only common bacterial species are listed in this table.

### Comparison of biomarkers in blood and IL-6 in bronchoalveolar lavage fluid among different community-acquired pneumonia severity categories of entire cohort of enrolled 118 cases

**FIGURE 1:**
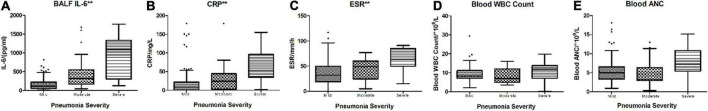
Boxplot of comparison in BALF IL-6, blood CRP, ESR, WBC count, and ANC across different severity categories. **(A)** BALF IL-6 (*p* = 0.000, < 0.01). **(B)** CRP (*p* = 0.001, < 0.01). **(C)** ESR (*p* = 0.004, < 0.01). **(D)** Blood WBC count (*p* = 0.109, > 0.01). **(E)** Blood ANC (*p* = 0.015, > 0.01). **p < 0.01.

There were significant differences in BALF IL-6 (*p* = 0.000, < 0.01), blood CRP (*p* = 0.001, < 0.01), and ESR (*p* = 0.004, < 0.01) levels across the three severity categories, whereas there were no significant differences in blood WBC count (*p* = 0.109, > 0.01) and ANC (*p* = 0.015, > 0.01) levels across the three categories (Figure 1). BALF IL-6, blood CRP, and ESR levels were elevated as the severity was upgraded from mild to severe categories, while blood WBC count and ANC showed no obvious trend with an increase in the severity.

### The performance of blood white blood cell, absolute neutrophil count, C-reactive protein, erythrocyte sedimentation rate, and bronchoalveolar lavage fluid interleukin 6 for discriminating severe from non-severe community-acquired pneumonia cases

**FIGURE 2:**
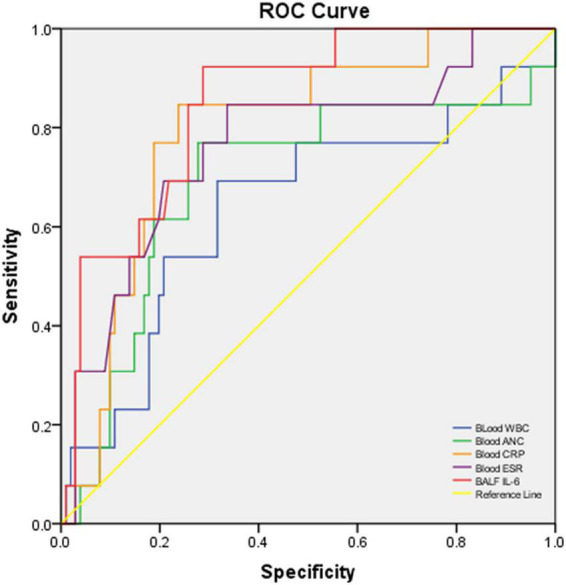
ROC curve of blood WBC, ANC, CRP, ESR, and BALF IL-6 for discriminating severe vs. non-severe CAP cases. The AUC and *p*-values of each blood biomarker and BALF IL-6 are listed in Table 2.

In the performance analysis of blood CRP, ESR, WBC count, ANC, and BALF IL-6 levels for discriminating severe CAP cases from non-severe cases with fair sensitivity and specificity, blood CRP (AUC: 0.799, *p*:0.001, < 0.05), ESR (AUC: 0.765, *p*:0.003, < 0.05), and BALF IL-6 (AUC: 0.847, *p* < 0.001, < 0.05) levels revealed better ability than blood WBC (AUC: 0.599, *p*: 0.095, > 0.05) and ANC (AUC: 0.672, *p*:0.053, > 0.05). Among the three better predictors, BALF IL-6 had best AUC [0.847 (0.748–0.946)] (Figure 2), sensitivity [0.917 (0.760–1.000)] and NPV [0.985 (0.956–1.000)] (Table 2).

**TABLE 2:** Performance characteristics of biomarkers or cytokines in blood/BALF to predict severe cases vs. non-severe cases with CAP.

Biomarker or cytokine	Threshold	AUC (95% CI)	*P*-value	Sensitivity (95% CI)	Specificity (95% CI)	PPV (95% CI)	NPV (95% CI)	LR + (95% CI	LR- (95% CI)
Blood WBC count	9.42	0.599 (0.412–0.785)	0.095	0.667 (0.400–0.933)	0.659 (0.562–0.757)	0.205 (0.078–0.332)	0.938 (0.878–0.997)	1.957 (1.197–3.200)	0.506 (0.224–1.141)
Blood ANC	6.41	0.672 (0.488–0.857)	0.053	0.750 (0.505–0.995)	0.714 (0.621–0.807)	0.257 (0.112–0.402)	0.956 (0.907–1.000)	2.625 (1.656–4.161)	0.350 (0.130–0.941)
Blood CRP	38.66	0.799* (0.663–0.935)	***0.001***	0.833 (0.622–1.000)	0.825 (0.742–0.908)	0.417 (0.219–0.614)	0.971 (0.930–1.000)	4.762 (2.778–8.162)	0.202 (0.057–0.719)
Blood ESR	46.50	0.765* (0.599–0.932)	***0.003***	0.833 (0.622–1.000)	0.671 (0.571–0.771)	0.263 (0.123–0.403)	0.966 (0.920–1.000)	2.530 (1.704–3.755)	0.249 (0.070–0.888)
BALF IL-6	263.10	0.847* (0.748–0.946)	**<*0.001***	0.917 (0.760–1.000)	0.725 (0.634–0.817)	0.306 (0.155–0.456)	0.985 (0.956–1.000)	3.337 (2.293–4.854)	0.115 (0.018–0.754)

PPV, positive predictive value; NPV, negative predictive value; LR + , positive likelihood ratio; LR-, negative likelihood ratio. *Good AUC (AUC > 0.600, p < 0.05) values are bold and italicized.

### The performance of blood white blood cell count, absolute neutrophil count, C-reactive protein, erythrocyte sedimentation rate, and bronchoalveolar lavage fluid interleukin 6 for discriminating mild vs. moderate–severe community-acquired pneumonia cases

**FIGURE 3:**
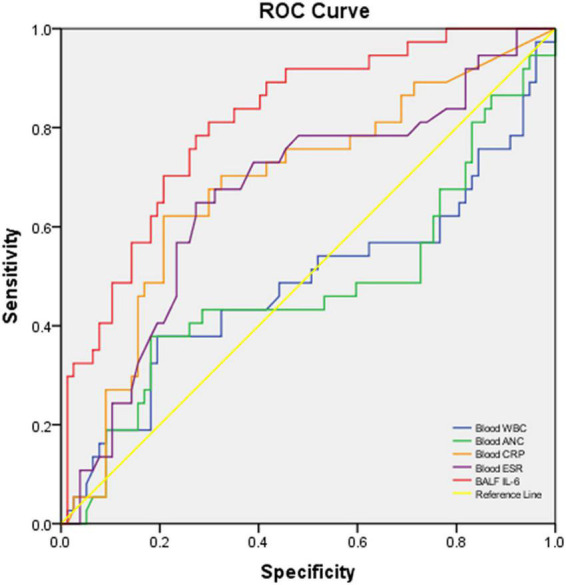
ROC curve of blood WBC, ANC, CRP, ESR, and BALF IL-6 for discriminating severe and moderate CAP cases vs. mild CAP cases. The AUC and *p*-values of each blood biomarker and BALF IL-6 are listed in Table 3.

In the performance analysis of blood CRP, ESR, WBC count, ANC, and BALF IL-6 for discriminating moderate-to-severe cases from mild cases with good sensitivity and specificity, blood CRP (AUC: 0.672, *p*:0.008, < 0.05), ESR (AUC: 0.641, *p* 0.025, < 0.05), and BALF IL-6 (AUC: 0.835, *p* < 0.001, < 0.05) had obvious ability than blood WBC (AUC: 0.494, *p* 0.923, > 0.05) and ANC (AUC: 0.488, *p* 0.852, > 0.05). Among the three good indicators, BALF IL-6 showed the best AUC [0.835 (0.755–0.915)] (Figure 3), sensitivity [0.839 (0.709–0.968)], PPV [0.591 (0.446–0.736)], NPV [0.915 (0.844–0.986)], and LR + [3.355 (2.185–5.151)] (Table 3) with nearly parallel specificity [0.750 (0.7650–0.850)] to that of blood CRP [0.794 (0.694–0.894)].

**TABLE 3:** Performance characteristics of biomarkers or cytokines in blood/BALF to predict moderate and severe vs. mild cases with CAP.

Biomarker or cytokine	Threshold	AUC (95% CI)	*P*-value	Sensitivity (95% CI)	Specificity (95% CI)	PPV (95% CI)	NPV (95% CI)	LR + (95% CI	LR-(95% CI)
Blood WBC count	11.62	0.494 (0.362–0.626)	0.923	0.378 (0.216–0.559)	0.792 (0.698–0.885)	0.444 (0.257–0.632)	0.750 (0.653–0.847)	1.858 (0.988–3.494)	0.774 (0.571–1.049)
Blood ANC	6.88	0.488 (0.356–0.621)	0.852	0.387 (0.216–0.559)	0.819 (0.731–0.908)	0.480 (0.284–0.676)	0.756 (0.661–0.852)	2.144 (1.106–4.157)	0.748 (0.554–1.010)
Blood CRP	27.46	0.672* (0.548–0.795)	***0.008***	0.586 (0.407–0.765)	0.794 (0.694–0.894)	0.567 (0.389–0.744)	0.806 (0.708–0.905)	2.841 (1.602–5.037)	0.521 (0.332–0.819)
Blood ESR	42.5	0.641* (0.517–0.766)	***0.025***	0.645 (0.477–0.814)	0.682 (0.569–0.794)	0.488 (0.335–0.641)	0.804 (0.699–0.908)	2.028 (1.307–3.146)	0.520 (0.315–0.860)
BALF IL-6	196.45	0.835* (0.755–0.915)	**<*0.001***	0.839 (0.709–0.968)	0.750 (0.650–0.850)	0.591 (0.446–0.736)	0.915 (0.844–0.986)	3.355 (2.185–5.151)	0.215 (0.095–0.485)

PPV, positive predictive value; NPV, negative predictive value; LR + , positive likelihood ratio; LR-, negative likelihood ratio. *Good AUC (AUC > 0.600, p < 0.05) values are bold and italicized.

### Blood biomarkers and bronchoalveolar lavage fluid interleukin 6 in different pathogen-detected categories

**FIGURE 4:**
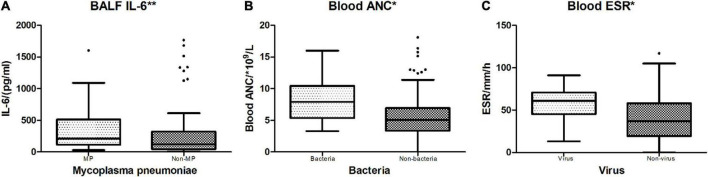
Indicators between different positive vs. non-positive pathogen groups. *p < 0.05; **p < 0.01. **(A)** MP vs. non-MP: Mycoplasma pneumoniae-detected cases vs. no Mycoplasma pneumoniae-detected cases. BALF IL-6 showed a significant difference between MP and non-MP groups (*p* = 0.000). **(B)** Bacteria vs. Non-bacteria: Bacteria-detected cases vs. no bacteria-detected cases. Blood ANC had a significant difference between Bacteria and Non-bacteria groups (*p* = 0.028). **(C)** Virus vs. non-virus: Virus-detected cases vs. no virus-detected cases. Blood ESR had a significant difference between Virus and Non-virus groups (*p* = 0.024).

Among the oral swab, blood, and BALF samples of 118 cases, there were 13 (11.0%) cases that had only virus detected, 24 (20.3%) had only MP detected, 13 (11.0%) had only bacteria detected, and 56 (47.5%) cases had no positive pathogen reported (Table 1). Comparison of blood WBC count, ANC, CRP, ESR, and BALF IL-6 levels across different pathogen-detected categories all revealed no significant differences (*p* > 0.05) (Table 4). In the comparison of biomarkers in blood and IL-6 in BALF between certain pathogen vs. non-certain pathogen categories (Table 5), BALF IL-6 levels had a significant difference between Mycoplasma pneumoniae (MP)-detected and non-MP-detected groups [MP: 210.2 (115.63, 512.33) pg/ml vs. Non-MP: 121.6 (43.45, 321.33) pg/ml, *p* = 0.000, < 0.05], whereas blood WBC count, ANC, CRP, and ESR levels showed no obvious difference between the two groups. Between bacteria vs. non-bacteria groups, blood ANC showed obvious difference when compared to the other parameters [bacteria: 7.93 (5.37, 10.44)*109/L vs. non-bacteria: 5.08 (3.33, 6.94)*109/L, *p* = 0.028, < 0.05]. Between virus and non-virus groups, ESR revealed significant differences, while the other parameters showed no significant differences [virus: 61 (45, 71) mm/h vs. non-virus: 37 (19, 58) mm/h, *p* = 0.024, < 0.05]. For BALF IL-6 levels in MP vs. non-MP, ANC in bacteria vs. non-bacteria, and ESR in virus vs. non-virus groups, the levels in the pathogen-positive group were all obviously higher than those in the non-pathogen groups (Figure 4).

**TABLE 4:** Comparison of blood WBC count, ANC, CRP, ESR, and BALF IL-6 levels across different pathogen-detected categories.

Biomarkers or cytokines	Pathogen detected categories				*P*-value
		
	MP	Bacteria	Virus	Others*	
Blood WBC/mean ± SD, *10^9^/L	8.76 ± 3.49	9.54 ± 4.63	8.64 ± 3.74	10.63 ± 5.61	0.234
Blood ANC/mean ± SD, *10^9^/L	7.68 ± 16.78	5.77 ± 4.50	5.32 ± 3.52	5.84 ± 4.25	0.939
Blood CRP/mean ± SD, mg/L	34.96 ± 60.30	19.40 ± 24.19	2.08 ± 40.79	29.44 ± 47.39	0.256
Blood ESR/mean ± SD, mm/h	41.61 ± 26.28	46.66 ± 25.69	52.85 ± 24.40	37.11 ± 27.02	0.147
BALF IL-6/mean ± SD, pg/ml	480.3 ± 647.0	423.5 ± 526.0	388.2 ± 578.3	394.3 ± 474.4	0.646

MP, Mycoplasma pneumonia. *Others category means other pathogen results except for MP, bacteria, and virus, including no pathogen detected.

**TABLE 5:** Comparison of biomarkers or cytokines between certain pathogen and non-pathogen groups.

*P*-value	Blood WBC	Blood ANC	Blood CRP	Blood ESR	BALF IL-6
MP vs. non-MP*	0.761	0.816	0.100	0.599	***0.000^#^***
Bacteria vs. non-bacteria**	0.380	***0.028^#^***	0.248	0.521	0.759
Virus vs. non-virus***	0.446	0.527	0.216	***0.024^#^***	0.385

*MP vs. non-MP: cases with Mycoplasma pneumoniae detected vs. cases without Mycoplasma pneumoniae detected, including bacteria, virus, and no pathogen detected. **Bacteria vs. Non-bacteria: cases with bacteria detected vs. cases without bacteria detected, including MP, virus, and no pathogen detected. ***Virus vs. Non-virus: cases with virus detected vs. cases without virus detected, including MP, bacteria, and no pathogen detected. ^#^Significant differences (p < 0.05) are bold and italicized.

### The correlation of blood biomarkers and bronchoalveolar lavage fluid interleukin 6 with hospitalization course

**FIGURE 5:**
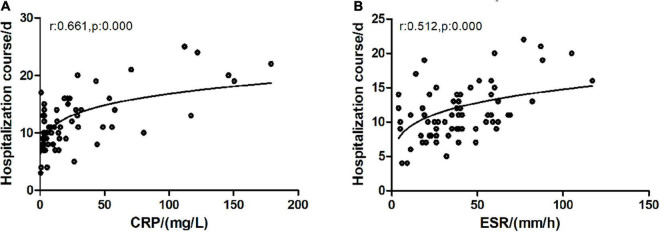
Correlation of CRP/ESR with hospitalization course. CRP, C-reactive protein; ESR, erythrocyte sedimentation rate. **(A)** C-reactive protein and hospitalization course exhibited a significantly positive correlation (Spearman test, *r* = 0.683, *p* = 0.000). **(B)** ESR and clinical course exhibited a significantly positive correlation (Spearman test, *r* = 0.512, *p* = 0.000).

In the correlation analysis of blood WBC, ANC, CRP, ESR, and BALF IL-6 with HC, CRP (*r* = 0.683, *p* = 0.000), and ESR (*r* = 0.512, *p* = 0.000), respectively, showed a significant correlation with HC (Figure 5), whereas BALF IL-6 (*r* = 0.061, *p* = 0.513), blood WBC (*r* = 0.055, *p* = 0.554), and ANC (*r* = 0.140, *p* = 0.132) showed no significant correlation with HC.

### Cytokines in bronchoalveolar lavage fluid across different severity categories

**FIGURE 6:**
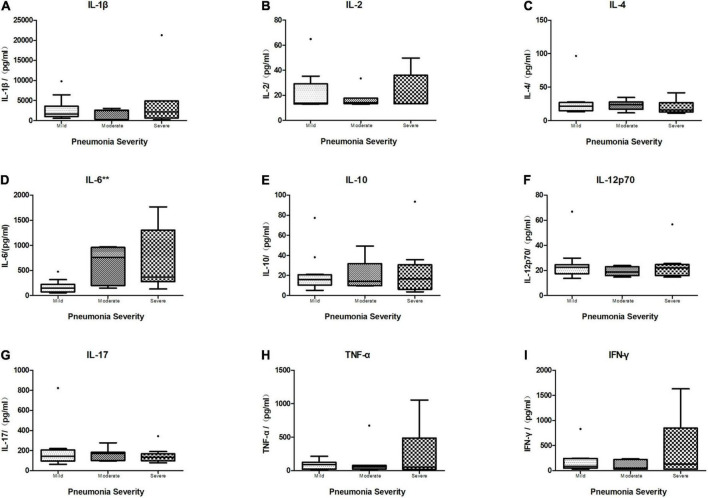
Boxplot of comparison of cytokine levels in BALF of different severity categories. The median cytokine concentration is represented by the middle line in each box. The lower and upper borders of the box represent the 25th and 75th percentiles, respectively. Only IL-6 **(D)** in BALF showed a significant difference within different severity categories (*N* = 28, Kruskal–Wallis test: *p* = 0.004, *p* < 0.01), while other cytokines revealed no significant difference within three groups (*N* = 28, Kruskal–Wallis test. **(A)** IL1β: *p* = 0.612, *p* > 0.05. **(B)** IL-2: *p* = 0.949, *p* > 0.05. **(C)** IL-4: *p* = 0.619, *p* > 0.05. **(E)** IL-10: *p* = 0.957, *p* > 0.05. **(F)** IL-12p70: *p* = 0.682, *p* > 0.05. **(G)** IL-17: *p* = 0.743, *p* > 0.05. **(H)** TNF-α: *p* = 0.784, *p* > 0.05. **(I)** IFN-γ: *p* = 0.834, *p* > 0.05). **p < 0.01.

Of the 28 children who had all the BALF cytokines tested, including IL-1β, IL-2, IL4, IL-6, IL-10, IL-12p70, IL-17, TNF-α, and IFN-γ (mean age 8.3 years, mean weight 30.8 kg, mean height 133.1 cm, and 32.1% were boys), 12 (42.9%) had mild CAP, 7 (25%) had moderate CAP, and 9 (32.1%) had severe CAP (Table 6.). Across the three severity categories, there were no differences in age (*p* = 0.746), sex (*p* = 0.729), and weight (*p* = 0.506). Only IL-6 in BALF showed a significant difference across the three groups (*N* = 28, Kruskal–Wallis test: *p* = 0.004, *p* < 0.01) (Figure 6), while there was no statistical difference in other BALF cytokine levels across the different severity groups (Figure 6).

**TABLE 6:** Characteristics of 28 cases with full BALF cytokines tested.

	*N* = 28
**Age, y, mean (SD)**	8.3 (3.6)
**Weight, kg, mean (SD)**	30.8 (15.1)
**Height, cm, mean (SD)**	133.1 (23.1)
**Males, *n* (%)**	9 (32.1)
**Fever, *n* (%)**	22 (78.6)
**Cough, *n* (%)**	24 (85.7)
**Complication, *n* (%)**	
Atelectasis	2 (7.1)
Hydrothorax	6 (21.4)
Pneumothorax	0 (0.0)
Sepsis, *n* (%)	6 (21.4)
**Pathogen detected, *n* (%)**	18 (64.3)
**Virus**	
Influenza	4 (14.3)
Coronavirus	3 (10.7)
Human metapneumovirus	1 (3.6)
Rhinovirus	2 (7.1)
Epstein-Barr virus	1 (3.6)
**Mycoplasma pneumoniae**	11 (39.3)
**Typical bacteria**	
Streptococcus pneumoniae	9 (32.1)
Haemophilus influenzae	5 (17.9)
Staphylococcus aureus	0 (0.0)
**Disease severity, *n* (%)**	
Mild	12 (42.9)
Moderate	7 (25.0)
Severe	9 (32.1)

**FIGURE 7:**
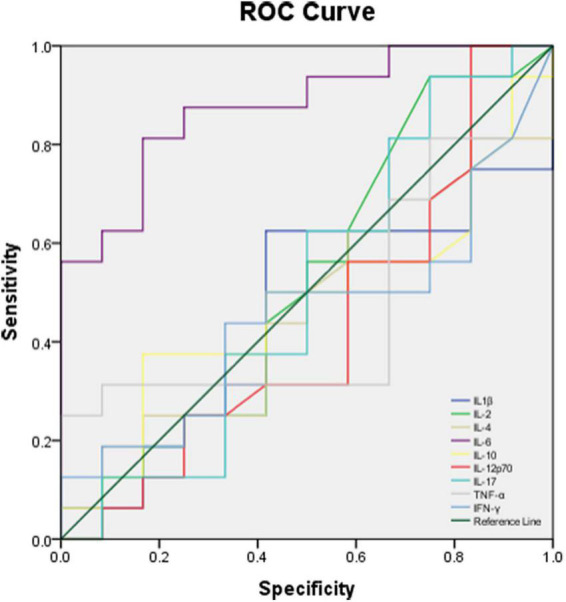
ROC curve of different BALF cytokines for predicting moderate and severe cases with CAP from mild cases.

The performance of BALF cytokines for predicting CAP severity showed that BALF IL-6 had a significant ability (AUC = 0.875, *P* = 0.004, < 0.01) compared to the other BALF cytokines (Table 7) in discriminating moderate and severe cases from mild cases, with limited ability for differentiating severe from non-severe cases (AUC = 0.731, *P* = 0.052). There was no significant performance of BALF IL-1β, IL-2, IL-4, IL-10, IL-12p70, IL-17, TNF-α, and IFN-γ in distinguishing severe to moderate from mild cases or severe cases from non-severe cases (Table 7). ROC curve of the performance of different BALF cytokines for predicting severe and moderate from mild cases showed better performance of BALF IL-6 among all the cytokines (Figure 7).

**TABLE 7:** Performance of cytokines in bronchoalveolar lavage fluid (BALF) for differentiating mild cases vs. moderate and severe cases across 28 cases.

	Mild vs. moderate + severe (12 vs. 16)*				Mild + moderate vs. severe (19 vs. 9)			
		
Cytokines	AUC	95% CI		*P*-value	AUC	95% CI		*P*-value
						
		Lower bound	Upper bound			Lower bound	Upper bound	
IL-1β	0.458	0.238	0.679	0.710	0.561	0.308	0.815	0.605
IL-2	0.521	0.292	0.749	0.853	0.538	0.306	0.770	0.749
IL-4	0.464	0.245	0.682	0.745	0.389	0.143	0.635	0.350
IL-6	***0.875***	***0.747***	***1.000***	0.001**	0.731	0.530	0.932	0.052
IL-10	0.479	0.260	0.698	0.853	0.465	0.194	0.736	0.768
IL-12p70	0.432	0.207	0.658	0.546	0.518	0.282	0.754	0.883
IL-17	0.505	0.275	0.736	0.963	0.430	0.204	0.656	0.555
TNF-α	0.471	0.245	0.697	0.798	0.544	0.278	0.809	0.712
IFN-γ	0.453	0.235	0.671	0.676	0.512	0.232	0.792	0.922

*For mild vs. moderate + severe cases, ROC curve is as shown in Figure 7. **Significant differences (p < 0.01) are bold and italicized (p-value not corrected for multiple corrected). AUC, area under the curve; CI, confidence interval; IL, interleukin; TNF, tumor necrosis factor; IFN, interferon.

### Blood and bronchoalveolar lavage fluid interleukin 6 in different severity categories

Among the 25 cases that had both blood and BALF IL-6 tested, blood IL-6 and BALF IL-6 levels revealed no significant correlation (correlation analysis Spearman test, *r* = 0.228, *p* = 0.272).

**FIGURE 8:**
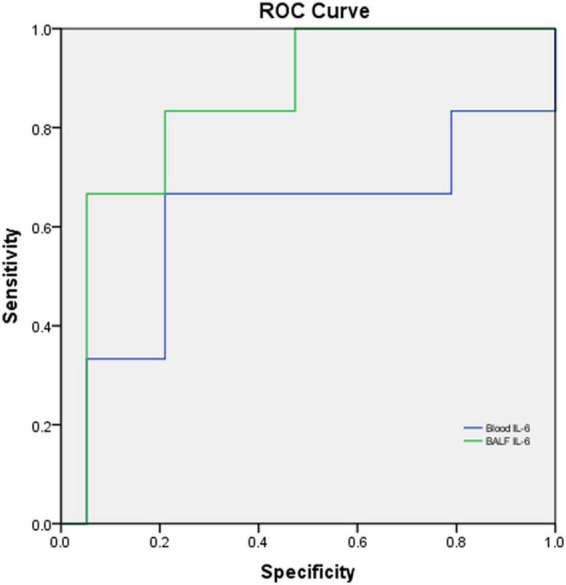
ROC curve of blood IL-6 and BALF IL-6 levels for differentiating severe and moderate cases from mild cases in CAP. *N* = 25, Blood IL-6: AUC = 0.614, *p* = 0.418; BALF IL-6: AUC = 0.851, *p* = 0.011, *p* < 0.05.

Among the 25 cases, 10 were severe and moderate cases, and 15 were mild cases. There were no significant differences between severe to moderate and mild categories in age (*p* = 0.323), sex (*p* = 0.912), and weight (*p* = 0.463). BALF IL-6 in severe and moderate vs. that in mild cases showed a significant difference (*p* = 0.027, < 0.05), while blood IL-6 (*p* = 0.290), CRP (*p* = 0.186), ESR (*p* = 0.497), PCT (*p* = 0.883), and HC (*p* = 0.303) had no significant difference between two categories, respectively. The performance of blood IL-6 and BALF IL-6 for differentiating severe and moderate cases from mild cases in CAP (Figure 8) showed that BALF IL-6 (AUC = 0.851, *p* = 0.011, *p* < 0.05) has a better discriminating ability in children with severe and moderate CAP from mild ones, while blood IL-6 levels (AUC = 0.614, *p* = 0.418) revealed limited ability for the discrimination.

## Discussion

Consistent with the previous literature, we found that blood WBC count and ANC had limited ability to predict CAP severity in children (11, 12). Studies on CRP and CAP severity in adults yield conflicting results. Some studies have revealed that CRP is associated with severe outcomes in pneumonia, such as mortality, complicated pneumonia, positive pressure ventilation, and inotropic support, whereas others have shown no association with severity or no value in adding CRP to the existing clinical severity scores (8, 10, 13–15).

A significant variation exists in the diagnosis and management of children with pneumonia. Although practice guidelines can assist with clinical decision-making, no validated and uniform severity criteria exist for children with CAP. In our study, we made the severity category rules according to the indication of IDSA guidelines (10). Moderate and severe categories were defined as mild cases with additional severe CAP criteria, such as tachypnea, apnea, altered mental status, hypotension, invasive mechanical ventilation, and hypoxemia. In the performance analysis of cytokines and biomarkers predicting severe and moderate cases of children with CAP from mild ones, both CRP and BALF IL-6 levels revealed significant ability. Although BALF IL-6 had better AUC, sensitivity, NPV, and LR + , what cannot be ignored is that CRP also showed relatively good prognosis ability than the rest of the biomarkers. It means that CRP was determined to have an association with and ability for predicting severe outcomes in children with CAP during hospitalization along with BALF IL-6.

Interleukin-6 acts as a major pro-inflammatory mediator for the induction of the acute phase response (16), leading to a wide range of local and systemic changes, including fever, leukocyte recruitment, activation, and hemodynamic effects. Considering the key role of IL-6 in mediating the acute phase response, its value as a prognostic biomarker in sepsis and various acute organ injuries has been extensively investigated in clinical and experimental studies. Plasma and/or bronchoalveolar levels of IL-6 have been identified as early biomarkers of lung injury and predictive factors of prolonged mechanical ventilation, organ dysfunctions, morbidity, and mortality in lung diseases (17, 18). Han et al. (19) enrolled 102 COVID-19 patients, classifying them into moderate, severe, and critical groups according to their symptoms with 45 control samples of healthy volunteers included, showing that COVID-19 patients have higher serum levels of cytokines (TNF-α, IFN-γ, IL-2, IL-4, IL-6, and IL-10) and CRP than control individuals, and within different severity COVID-19 groups, serum IL-6 and IL-10 levels are found to be predictive of disease severity. Nowadays, IL-6 has also been searched from signaling to drug discovery (20).

Given that previous studies in adults demonstrated IL-6 to have predicting ability in adult diseases, data in children with CAP are limited. In our study, we investigated the ability of different BALF cytokines to predict disease severity in hospitalized children with CAP, and the results revealed that IL-6 was good at predicting CAP severity when compared to the other markers. Next, we concluded that BALF IL-6 had no correlation with blood IL-6, and BALF IL-6 showed a significant difference in different severity categories in hospitalized children with CAP and better performance in predicting CAP severity in hospitalized children compared to blood IL-6.

Recent studies demonstrated that cytokines are the effect elements of numerous inflammation cells or infection conditions. Some studies revealed that interleukins (e.g., IL-6, IL-8, etc.) in blood and BALF can predict infections caused by opportunistic pathogens or severe outcomes in infection diseases (5, 21). However, Speck et al. (22) suggested that cytokines in bronchoalveolar lavage are of minor value to diagnose complications following lung transplantation. They hinted that cytokines could not work as predictors in some special immunity conditions of infectious diseases. The studies relating to the predicting ability of cytokines seemed to be more focused on rare disease conditions. So, in our study, we investigate the predicting ability of cytokines in children with CAP. The study revealed positive results that IL-6, compared to other cytokines in BALF, seemed to have a better ability in predicting CAP severity in children with a higher AUC and fair sensitivity and specificity, while blood IL-6 levels revealed no significant association with CAP severity. However, we still cannot reach definitive conclusions about the good performance of IL-6 due to the limitation of sample size and retrospective analysis approach. More further studies are needed to support this emerging trend.

It is worth noting that BALF was more difficult to obtain than the blood samples in the majority of medical facilities. It seemed that CRP and ESR levels in blood samples are more practical and economical to be used in predicting CAP severity and poor outcomes in hospitalized children. Nevertheless, an undeniable fact is that more number of medical facilities will be qualified to perform bronchoscopy lavage management for children with CAP, who were eligible for this according to the guidelines. In addition, bronchoalveolar lavage, indeed, could obviously improve CAP outcomes and shorten the HC (23), especially in our daily clinical experience. Recently, BALF IL-6 could be used in severe cases eligible for bronchoalveolar lavage management guidelines as a major marker in predicting severe CAP, to help more precise prognosis and more timely management or precautions in children with severe CAP.

As for the pathogen spectrum and proportion of severe cases, obvious variations exist across different studies in different regions. In the United States, the pathogens detected in children with CAP were mainly viruses, with a minority of MP and bacteria and a relatively low severity proportion (2). Several case-control or descriptive studies in European countries, mainly in high-income countries, have reported that vaccination, especially with the 13-valent PCV (PCV13), has reduced the incidence and severity of pneumonia in children and complications like empyema (24–26). A novel study about children with CAP in China reported a consistent conclusion with our study that MP and bacterial infections were nearly equal to virus infections, and severe cases were not so rare like developed countries (27). In our study, we still have a relatively higher percentage of MP and bacteria-related pneumonia in children with CAP and more severe cases than in European countries and the USA, consistent with the literature in our country or nearby regions of Asia.

Our study has several important strengths. We had retrospectively investigated major cytokines in BALF in hospitalized children with CAP, whereas previous literature mostly focused on adult studies, to investigate which was better for predicting CAP severity and complications in children. We were able to first investigate the role of BALF IL-6 in hospitalized children with CAP for predicting the poor outcome and the occurrence of underlying complications, which is an important step because children with complicated CAP often come without typical manifestations or signs, leading to rapid aggravations within a short period. BALF IL-6 could help in the sensitive prediction of CAP-associated severe complications in hospitalized children on time with the help of conventional biomarkers of blood (CRP and ESR), so that clinicians could provide adequate management before the manifestation of poor outcomes. Our study revealed that the conventional biomarkers, such as WBC, ANC, CRP, and ESR, are not helpful in discriminating pathogen categories, conflicting with the outmoded concepts of pathogen prediction.

Our study also has several limitations. First, this study is a retrospective study. We could not avoid bias by study design, such as epidemic pathogen, although we had already set exclusion criteria to avoid predisposal effects and special immunosuppression conditions. Second, data in this study were collected in one single center, so the results may not be generalizable. Validation across centers is important. Third, the performance of cytokines for predicting CAP severity was limited to case number (*n* = 28), and the performance of blood and BALF IL-6 for predicting disease severity was limited as well (*n* = 25). Further investigations of a larger cohort are needed, and we could add IL-6 in induced sputum investigation for easier diagnosis. As a limitation, although we performed BAL for CAP with the support of national guidelines, it should be noted that global centers choose not to perform BAL routinely for CAP. We realize that bronchoalveolar lavage, indeed, could obviously improve CAP outcomes with safety and reasonable cost performance (28–30), which we are collecting related data to support in the near future.

In summary, our results suggest that conventionally measured biomarkers, including CRP and ESR, in addition to BALF IL-6, are useful for predicting disease severity in children with CAP. Given their high AUC, sensitivity, and fair specificity, these markers, especially BALF IL-6, could be more helpful in ruling out severe outcomes. BALF IL-6, CRP, and ESR may help in diagnosing single MP, bacteria, or virus infection, respectively. Further research with a larger cohort is required to understand if there are specific clinical situations (e.g., severe outcomes) in which these predictors improve current predictive ability, allowing for targeted interventions in high-risk children.

## Data availability statement

The original contributions presented in this study are included in the article/supplementary material, further inquiries can be directed to the corresponding author/s.

## Ethics statement

The studies involving human participants were reviewed and approved by the Qilu Hospital of Shandong University Institutional Review Board. Written informed consent from the participants’ legal guardian/next of kin was not required to participate in this study in accordance with the national legislation and the institutional requirements.

## Author contributions

YZ initiated the study, participated in the design and coordination, and drafted the manuscript. WZ and HN contributed equally to data management. JL provided the statistical support. XJ and FL helped to initiate the study and edit the manuscript. All authors contributed to the article and approved the submitted version.
